# Dermoscopy of acute radiation-induced dermatitis in patients with head and neck cancers treated with radiotherapy

**DOI:** 10.1038/s41598-023-42507-1

**Published:** 2023-09-21

**Authors:** Aleksandra Pilśniak, Anastazja Szlauer-Stefańska, Andrzej Tukiendorf, Tomasz Rutkowski, Krzysztof Składowski, Grażyna Kamińska-Winciorek

**Affiliations:** 1 Department of Internal Medicine, Autoimmune and Metabolic Diseases, Faculty of Medical Sciences in Katowice, Medical University of Silesia, Katowice, Poland; 2 Department of Bone Marrow Transplantation and Onco-Hematology, Maria Sklodowska-Curie National Research Institute of Oncology (MSCNRIO), Gliwice, Poland; 3 Institute of Health Sciences, Opole University, Opole, Poland; 4 Inpatient Department of Radiation and Clinical Oncology, Maria Sklodowska Curie National Research Institute of Oncology (MSCNRIO), Gliwice, Poland; 5 Department of Bone Marrow Transplantation and Onco-Hematology, Skin Cancer and Melanoma Team, Maria Sklodowska-Curie National Research Institute of Oncology (MSCNRIO), Wybrzeże Armii Krajowej 15, 44-101 Gliwice, Poland

**Keywords:** Optical imaging, Radiotherapy, Head and neck cancer, Physical examination, Quality of life, Adverse effects, Radiotherapy

## Abstract

Head and neck cancer (HNC) was the seventh most common cancer in the world in 2018. Treatment of a patient may include surgery, radiotherapy (RT), chemotherapy, targeted therapy, immunotherapy, or a combination of these methods. Ionizing radiation used during RT covers relatively large volumes of healthy tissue surrounding the tumor. The acute form of radiation-induced dermatitis (ARD) are skin lesions that appear usually within 90 days of the start of RT. This is a prospective study which compares 2244 dermoscopy images and 374 clinical photographs of irradiated skin and healthy skin of 26 patients at on average 15 time points. Dermoscopy pictures were evaluated independently by 2 blinded physicians. Vessels in reticular distribution, white, yellow or brown scale in a patchy distribution, perifollicular pigmentation and follicular plugs arranged in rosettes were most often observed. For these dermoscopic features, agreement with macroscopic features was observed. Two independent predictors of severe acute toxicity were identified: gender and concurrent chemotherapy. Knowledge of dermoscopic features could help in the early assessment of acute toxicity and the immediate implementation of appropriate therapeutic strategies. This may increase the tolerance of RT in these groups of patients.

## Introduction

Head and neck cancer (HNC) is one of the most common cancers and continues to be a significant challenge in clinical practice^1^. Each year, around 800 thousand patients worldwide develop HNC, and approximately half of them die from the disease^1^. Head and neck cancers are more than twice as common in men than in women^2^. According to the definition of the American Joint Committee on Cancer (AJCC), this group of neoplasms includes those originating from the mucosa of the oral cavity, pharynx, larynx, paranasal sinuses, or major and minor salivary glands^3^. The most common histological type of neoplasm in this area is squamous cell carcinoma^4^. Treatment is multimodal and depends on many tumor and patient-related factors and usually includes surgery, radiotherapy (RT), and chemotherapy which are often combined ^5^. The prognosis depends mainly on the stage of the disease. Despite aggressive multimodal treatment strategies, poor results are still observed. The 5-year survival is only 40–50%^6^. Ionizing radiation used during RT covers relatively large volumes of healthy tissue surrounding the tumor because irradiated volume extends beyond gross tumor volume to clinical tumor volume 1 (CTV1) covering the potential microscopic spread of the tumor and to planned target volume 1 (PTV1) that cover margin dedicated to technical aspects of radiotherapy. A typical therapeutic dose is usually from the range of 66–74 Gy in fractions of 2.0 Gy or even higher doses (e.g. 81.6 Gy in fractions of 1.2 Gy)^7^. To improve local control and reduce the toxic effect, fractionation approaches can be divided into hyperfractionation and accelerated fractionation^8^. Early skin reactions to RT can occur within the first 24 h of starting RT but usually begin within a few days or even weeks from the beginning of RT. The acute form of radiation-induced dermatitis is skin lesions that appear within 90 days of RT begining^9^. Acute radiation-dermatitis (ARD) is responsible for discomfort, pain, aesthetic changes, and may reduce patient’s quality of life. Intense ARD may even cause the need to reduce the RT dose or stop RT for some time to heal the mucosal or skin reaction. Both situations increase the risk of treatment failure^10–12^. Clinical evaluation of radiation-induced dermatitis is not standardized, and multiple clinical scales have been described. The most frequently used are the scale of the Radiation Therapy Oncology Group and the European Organization for Research and Treatment of Cancer (RTOG/EORTC)^13^, Common Terminology Criteria for Adverse Events (CTCAE) v. 5.0^14^, the Late Effects Normal Tissue Task Force-Subjective scale, and the Objective, Management, Analytic scale (LENT SOMA)^15,16^. According to the RTOG/EORTC classification^13^, in grade I, we can observe follicular, faint, or dull erythema, epilation, dry desquamation, and decreased sweating. Grade II occurs when the following features are observed: tender or bright erythema, patchy moist desquamation, and moderate edema. In grade III of ARD, there is confluent, moist desquamation other than skin fold and pitting edema may occur. In grade IV, the presence of ulceration, and hemorrhage necrosis is stigmatized. Grade V is known as death^13^. Dermoscopy is a recognized diagnostic method combining clinical and pathological examination. There is no data concerning the evaluation of dermoscopic features of ARD in current literature. The innovative application of dermoscopy in the assessment of ARD may allow the standardization of its clinical evaluation. Consequently, a proper assessment of the severity of ARD skin damage will make it possible to decide how to manage the patients who undergo RT due to HNC.

## Materials and methods

This study aimed to assess dermoscopic features of ARD among patients with HNC qualified for RT, with a subsequent analysis of clinical and dermoscopic patterns of the treated and control areas, based on obtained macroscopic and dermoscopic photographs of ARD for further comparison.

### Patients

The study group consisted of 26 patients who underwent RT due to HNC (24 squamous cell carcinomas, one lymphoepithelial carcinoma, and one undifferentiated nasopharyngeal carcinoma) at the Maria Skłodowska-Curie National Research Institute of Oncology, Gliwice Branch, between September 2020 and March 2021.The inclusion criteria were age > 18 years, radical treatment signed consent. Patients treated with biological drugs (bio radiodermatitis), and with active dermatoses that could affect the clinical and dermoscopic picture of the examined skin area under observation were excluded from the study. Details of the patients’ clinical and histopathological characteristics, the location of the tumor, are shown in Table 1. The control group consisted of skin regions not exposed to ionizing irradiation from the same patients (748 images).

**Table 1:** Clinical characteristics, location of the tumor, and histopathological type of the group of observed patients.

Median age (range)	Gender (M/F)	Location of tumor	Histopathological type (WHO) classification
61 (34–74)	21/5	Lower (3), middle (7), and upper (2) pharynx; epiglottis (1), glottis (1); larynx (8); palatine tonsil (2), an alveolar triangle of palatine tonsil (1); metastasis to the lymphatic system of the neck from an unknown primary site (1)	Carcinoma planoephiteliale (24) Lymphoepitelial carcinoma (1) Undifferentiated nasopharyngeal carcinoma (1)

### Treatment

In seven cases, induction chemotherapy (indCHT) prior to radiochemotherapy (CHRT) was given; CHRT and RT alone was applied in 12 and four patients, respectively. The median total RT dose was 70 Gy (50–72 Gy) given in 25–40 daily fractions. Radiotherapy was delivered for over 7 weeks by incorporating five fractions per week combined with chemotherapy (CHT) (cisplatin, 100 mg/m^2^ days (d) 1, 22, 43) or as a concomitant boost (CB) with seven fractions per week without CHT. Clinical target volume 1 (CTV1) included a primary tumor and involved lymph node groups with a margin. Clinical target volume 2 (CTV2) included CTV1 and areas at risk of harboring microscopic spread of primary tumor and elective lymph node groups. All patients were treated with doses of 70 Gy in 35 fractions (2.0 Gy/fraction) for over 7 weeks or 70.2 Gy in 39 fractions (1.8 Gy/fraction) for over five and a half weeks to the primary target. Doses to the elective target were 50 Gy in 25 fractions (2.0 Gy/fraction) or 54 Gy in 30 fractions (1.8 Gy/ fraction). Induction chemotherapy consisted of two to three cycles of TPF (docetaxel 75 mg/m^2^, cisplatin 75 mg/m^2^, d1 and 5-fluorouracil 750 mg/m^2^ d1–5) or PF (cisplatin 100 mg/m^2^, d1 and 5-fluorouracil 1000 mg/m^2^ d1–5).

### Clinical and dermoscopic evaluation

Patients were evaluated clinically and dermoscopically on average at 15-time points—at the beginning of the study (prior to RT), then every other day until the end of the hospitalization: in 1, 2, 4, 6, 8, 10, etc. Each patient was assessed in the same symmetric four areas (right and left cervical areas, right and left submandibular areas) exposed to ionizing irradiation and in two control areas (right and left retroauricular regions). During the entire period, 374 observations were made in all patients; during each, four dermoscopic photos of the irradiated area and two photos of the non-treated area were taken. A total of 2244 dermoscopic photographs and 374 clinical photographs were recorded. Out of them, 1496 photographs represented the investigated areas exposed to irradiation. Clinical evaluation was performed in line with the RTOG/EORTC radiation-induced dermatitis scale (I–V). The presence of erythema, epilation, dry desquamation, moist desquamation, moderate edema, pitting edema, ulceration, hemorrhages, and necrosis was assessed. Dermoscopic findings were described in line with the consensus of experts in non-neoplastic dermatoses on behalf of the International Dermoscopy Society (IDS) by Errichetti et al.^17^. The presence or absence of 31 clinical features was described, including vessels (morphology and distribution); scale (color and distribution); follicular findings (follicular plugs, follicular red dots, perifollicular white color, follicular pigmentation); other structures (color and morphology); and specific clues. Dermoscopic assessment of skin lesions was performed using the DermLiteFoto dermoscope (*3Gen, LLC*, *San Juan Capistrano*, CA, USA) at tenfold magnification. Dermoscopy was performed by a medical doctor experienced in dermoscopy (A. P.). Dermoscopic images were then independently analyzed by two dermoscopists (A. P. and A. S.-S.), blinded to any patient/protocol data. When there was a discrepancy between them, the third dermoscopist (G. K.-W.) made the final decision regarding the description.

### Statistical analysis

A photographic database of 2244 dermoscopic photographs and 374 clinical photographs was analyzed in the final statistical assessment. Concordance based on Cohen’s κ coefficient in the assessment of dermoscopic and macroscopic photographs between two independent observers in 89% of the results was greater than or equal to 0.9. In particular, the value of κ ranges between − 1 and + 1 (κ equal to + 1 implies a perfect agreement between the two ratings, while that of − 1 implies perfect disagreement; if κ assumes the value 0, then this implies that there is no relationship between the two ratings, and any agreement or disagreement is random). Univariate and multivariate binary logistic regression was applied to evaluate the impact of the RT fractions on binary skin diagnostic outcomes. In turn, to estimate the influence of the collected risk factors on the observed dermoscopic features, a multivariate ordinal logistic model was used. The statistical outcomes were expressed by a classical odds ratio (OR) with a 95% confidence interval (CI); a p value of < 0.05 was considered statistically significant. Due to repeated measures with consecutive RT fractions for each patient, the regressions were extended for random effects. The statistical outcomes were expressed by a classical odds ratio (OR) together with a 95% confidence interval (CI 95%) and a p value.

### Ethical approval

The authors have received approval from the local ethics committee of the National Research Institute of Oncology (reference number KB/430-44/19). The study was conducted in accordance with the Helsinki Declaration of 1964, and its later amendments. All subjects provided informed consent to participate in the study as well as for publication.

## Results

There were oral cavity carcinoma, oropharyngeal carcinoma, hypopharyngeal carcinoma, laryngeal carcinoma nasopharyngeal carcinoma and neck lymph nodes tumor as a metastatic cancer from unknown primary in 1, 8, 3, 10, 3 and 1 patients, respectively. There were five women and 21 men with the mean age of 60.5 years (range 34–74) in this group.

**Figure 1:**
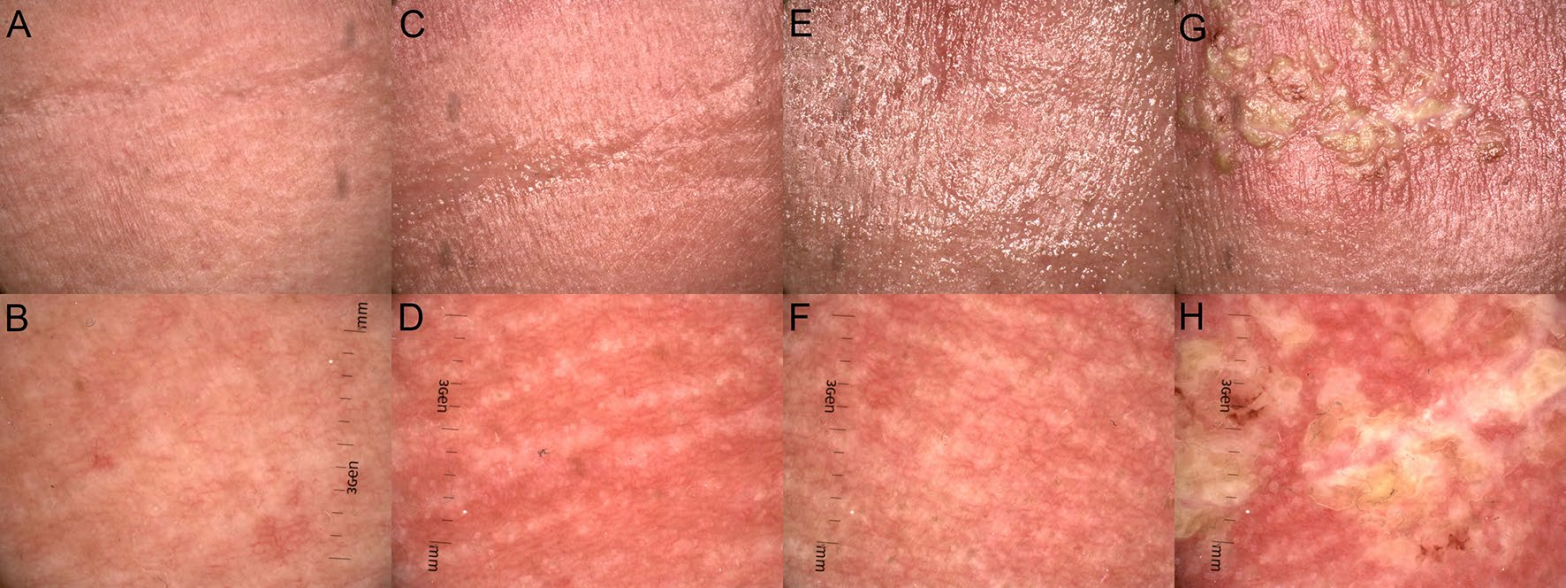
Macroscopic images (**A**,**C**,**E**,**G**) of ARD in grades (G) from G1 to G4, clinically assessed in line with RTOG criteria^13^ and dermoscopic findings (**B**,**D**,**F**,**H**) described in line with the consensus of experts in non-neoplastic dermatoses on behalf of the International Dermoscopy Society in one of the patients observed during the course of the RT treatment. (**A**) Faint erythema (G1); (**B**) dermoscopic image (G1) of ARD reveals linear branched and linear curved vessels in clustered distribution and white structureless areas; (**C**) bright erythema, epilation, moist desquamation and moderate edema (G2); (**D**) dermoscopic image of ARD (G2) shows linear branched and linear curved vessels in reticular distribution of vessels, and follicular plugs arranged in rosettes; (**E**) bright erythema, epilation, confluent moist desquamation and pitting edema (G3); (**F**) dermoscopic image (G3) with linear branched vessels in reticular distribution, perifollicular pigmentation and follicular plugs arranged in rosettes; (**G**) ulceration in ARD (G4); (**H**) dermoscopic image (G4) reveals linear branched vessels in reticular distribution, white, yellow, patchy scale.

All patients (26) observed during the course of RT developed ARD. The highest noted grade according to RTOG/EORTC, at the end of the RT treatment, was grade II in 14 patients, grade III in 10 patients, and the remaining two developed grade IV ARD. Grade I was observed in the first week (on average on Day 4.69) (Fig. 1A), grade II in the third week of the follow-up (Day 20.69) (Fig. 1C), grade III in the 6th week of the follow-up (Day 37.81) (Fig. 1E), and grade IV in the 5th week of the follow-up (Day 34.66) (Fig. 1G). The percentage occurrence of dermoscopic features depending on the grade of radiation-induced dermatitis per RTOG/EORTC^13^ is presented in Table 2 and Fig. 1B,D,F,H (Table 2).

**Table 2:** Percentage share (%) of dermoscopic non-neoplastic features^17^ depending on the grade of radiodermatitis in line with RTOG/EORTC^13^.

Dermoscopic features	Prior RT (%)	RTOG I (%)	RTOG II (%)	RTOG III (%)	RTOG IV (%)
Vessels morphology					
Dotted	61.5	62.0	71.7	59.3	100
Linear (without bends or branches)	2.6	0.7	0.6	0	0
Linear with branches	87.2	95.6	97.0	100.0	100
Linear curved	92.3	97.8	98.8	100.0	100
Vessels distribution					
Uniform	0	0	0	0	0
Clustered	35.9	79.6	59.6	70.4	33.33
Peripheral	0	0	0	0	0
Reticular	0	30.7	62.7	70.4	66.67
Unspecific	97.4	52.6	35.5	18.5	33.33
Scale color					
White	0	21.9	48.2	70.4	100
Yellow	0	8.8	33.7	63.0	100.0
Brown	0	4.4	34.9	63.0	66.7
Scale distribution					
Diffuse	0	0	0	0	0
Central	0	0	0	0	0
Peripheral	0	0	0	0	0
Patchy	0	24.8	74.1	92.6	100.0
Follicular findings					
Follicular plugs arranged in rosettes	0	8.0	54.2	44.4	100.0
Follicular red dots	0	0	0	0	0
Perifollicular white color	38.5	21.9	41.0	18.5	33.3
Perifollicular pigmentation	12.8	27.0	61.5	44.4	66.7
Other structures					
White structureless	46.2	59.1	47.0	63.0	66.7
Brown structureless	0	0	0	0	0
Yellow structureless	0	0	0	0	0
White dots or globules	0	0	0	0	0
Brown dots or globules	20.5	26.3	19.3	3.7	0
Yellow dots or globules	0	0	0	0	0
White lines	35.9	54.0	44.0	14.8	0
Brown lines	25.6	37.2	25.3	14.8	0
Yellow lines	7.7	1.5	1.8	0	0

Summary of dermoscopic findings: vessels in each grade of ARD were polymorphic. The arrangement of the vessels was also heterogeneous, and there was no typical arrangement for a particular grade of ARD. In healthy skin, we did not observe vessels in reticular distribution, but their presence was detected in every degree of ARD. Unspecific distribution was more common in healthy skin than in ARD. In each grade of ARD, a patchy scale was observed and the frequency of scale occurrence increased with the grade of development according to RTOG without characteristic color was observed. However, the incidence of scale increases with the degree of development in RTOG (Fig. 1H). Moreover, a feature present in all grades but not observed in healthy skin was follicular plugs arranged in rosettes.

Statistically significant results are underlined in bold in Table 3. A relationship between the observed dermoscopic and clinical features was checked using κ coefficient (Table 3).

**Table 3:** Level of agreement between the presence of selected dermoscopic features^17^ and clinical features^13^ in ARD assessed with values of κ statistics.

Dermoscopic features	Clinical features							
	Faint erythema	Bright erythema	Epilation	Dry desquamation	Moist desquamation	Moderate edema	Pitting edema	Ulceration
Dotted vessels	− 0.04	0.07	− 0.02	0.03	0.00	0.02	0.00	− 0.02
Linear vessels	0.00	− 0.01	− 0.01	− 0.02	0.01	0.00	− 0.01	− 0.01
Linear witch branches vessels	0.00	0.03	0.04	0.01	0.00	0.00	0.01	0.00
Linear curved vessels	0.00	0.02	0.02	0.00	0.01	0.01	0.00	0.00
Clustered vessels	**0.18**	− 0.09	− **0.16**	0.05	− **0.15**	− **0.15**	0.00	0.01
Reticular vessels	− **0.21**	**0.42**	**0.35**	0.02	**0.30**	**0.22**	**0.09**	− 0.02
Unspecific vessels	0.06	− **0.29**	− **0.21**	0.05	− **0.21**	− **0.18**	− **0.09**	− **0.03**
White scale	− **0.19**	**0.34**	**0.29**	**0.23**	**0.19**	**0.17**	**0.15**	**0.08**
Yellow scale	− **0.23**	**0.32**	**0.32**	**0.42**	**0.23**	**0.23**	**0.24**	**0.13**
Brown scale	− **0.26**	**0.34**	**0.34**	**0.14**	**0.42**	**0.45**	**0.23**	**0.07**
Patchy scale	− **0.31**	**0.54**	**0.53**	**0.23**	**0.35**	**0.41**	**0.14**	**0.04**
Follicular plugs arranged in rosettes	− **0.35**	**0.47**	**0.46**	**0.15**	**0.44**	**0.52**	**0.09**	0.01
Perifollicular white color	− **0.16**	**0.11**	0.06	**0.11**	− 0.08	0.07	− 0.05	− 0.03
Perifollicular pigmentation	− **0.20**	**0.32**	**0.45**	**0.16**	**0.12**	**0.20**	0.02	− 0.02
White structureless	**0.10**	− 0.07	0.00	− 0.04	0.03	− 0.02	0.03	**0.03**
Brown dots or globules	**0.12**	− **0.09**	− **0.09**	− 0.07	− **0.14**	− 0.02	− **0.11**	− 0.04
White lines	**0.15**	− **0.13**	− **0.13**	**0.10**	− **0.24**	− **0.13**	− **0.11**	− **0.04**
Brown lines	**0.12**	− **0.13**	− 0.06	− 0.03	− **0.11**	− **0.14**	− 0.07	− 0.04
Yellow lines	− 0.01	− **0.03**	0.01	− 0.04	− 0.04	0.02	− 0.03	− 0.02

The agreement between dermoscopic and clinical features was 0.03–0.54 and bright erythema, epilation, dry and moist desquamation, moderate edema, and dermoscopic features such as vessels in reticular distribution, white, yellow, brown scale and patchy scale distribution, follicular plugs arranged in rosettes and perifollicular pigmentation. Negative results mean incompatibility: when a given macroscopic feature is present, the dermoscopic feature is not present. In the next step, dermoscopic and clinical features were analyzed in terms of the influence of time, age, gender, induction chemotherapy, concurrent chemotherapy, total radiation dose, fractional dose, tumor location, as well as the histopathological diagnosis during the whole RT treatment on the skin diagnostic outcomes using logistic regression. The statistically significant relationships between clinical features and possible ARD risk factors—time, age, gender, indCHT, concurrent CHT, and fractional dose—are expressed by odds ratios reported in Table 4 whereas OR is a measure of association between radiation exposure and a clinical outcome; OR > 1 indicates the increased occurrence of any event, while OR < 1 a protective exposure) (Table 3).

**Table 4:** Statistically significant ORs (p < 0.05) of the influence of clinical data on the occurrence of dermoscopic and macroscopic features in ARD (univariate and multivariate logistic regression).

	Univariate analysis		Multivariate analysis	
Dermoscopic features				
Reticular vessels				
Time	1.08 (1.06–1.10)	** < 0.0001**	1.10 (1.08–1.12)	** < 0.0001**
Age	0.97 (0.94–0.99)	**0.0031**	0.95 (0.92–0.98)	**0.0009**
Gender	0.34 (0.18–0.60)	**0.0001**	0.15 (0.07–0.33)	** < 0.0001**
Induction chemotherapy	2.94 (1.85–4.74)	** < 0.0001**	5.90 (3.10–11.7)	** < 0.0001**
Radiochemotherapy	1.83 (1.12–3.03)	**0.0149**	0.47 (0.23–0.96)	**0.0379**
Unspecific vessels				
Time	0.95 (0.93–0.96)	** < 0.0001**	0.94 (0.93–0.96)	** < 0.0001**
Age	1.03 (1.01–1.06)	**0.0157**	1.03 (1.00–1.05)	0.0505
Gender	1.97 (1.17–3.38)	**0.0110**	2.15 (1.21–3.86)	**0.0085**
White scale				
Time	1.06 (1.04–1.08)	** < 0.0001**	1.07 (1.05–1.09)	** < 0.0001**
Gender	0.14 (0.06–0.31)	** < 0.0001**	0.11 (0.04–0.25)	** < 0.0001**
Yellow scale				
Time	1.07 (1.05–1.09)	** < 0.0001**	1.08 (1.05–1.10)	** < 0.0001**
Gender	0.23 (0.08–0.51)	**0.0001**	0.20 (0.07–0.48)	**0.0001**
Induction chemotherapy	0.47 (0.25–0.84)	**0.0095**	0.34 (0.17–0.66)	**0.0011**
Brown scale				
Time	1.09 (1.07–1.12)	** < 0.0001**		
Patchy scale				
Time	1.11 (1.09–1.14)	** < 0.0001**	1.13 (1.10–1.16)	** < 0.0001**
Gender	0.27 (0.15–0.47)	** < 0.0001**	0.12 (0.05–0.25)	** < 0.0001**
Follicular plugs arranged in rosettes				
Time	1.12 (1.09–1.15)	** < 0.0001**	1.13 (1.10–1.16)	** < 0.0001**
Age	0.97 (0.94–0.99)	**0.0053**	0.97 (0.94–1.01)	0.1226
Gender	0.51 (0.27–0.94)	**0.0293**	0.31 (0.14–0.66)	**0.0020**
Radiochemotherapy	1.89 (1.10–3.37)	**0.0207**	1.12 (0.54–2.34)	0.7650
Perifollicular pigmentation				
Time	1.06 (1.04–1.08)	** < 0.0001**	1.07 (1.05–1.09)	** < 0.0001**
Age	1.03 (1.00–1.06)	**0.0194**	1.02 (0.99–1.05)	0.1250
Radiochemotherapy	0.48 (0.30–0.78)	**0.0029**	0.49 (0.26–0.95)	**0.0334**
Fraction dose	0.02 (0.00–0.18)	**0.0005**	0.04 (0.00–0.54)	**0.0143**
Clinical features				
Follicular erythema				
Time	0.89 (0.74–0.98)	**0.0158**	0.89 (0.73–0.99)	**0.0276**
Age	1.30 (1.05–1.69)	**0.0104**	1.31 (1.01–1.82)	**0.0417**
Radiochemotherapy	0.13 (0.01–0.81)	**0.0290**	0.42 (0.04–2.71)	0.3654
Faint erythema				
Time	0.94 (0.92–0.95)	** < 0.0001**	0.93 (0.92–0.95)	** < 0.0001**
Gender	1.85 (1.10–3.14)	**0.0212**	2.18 (1.23–3.92)	**0.0081**
Tender erythema				
Radiochemotherapy	0.06 (0.00–0.77)	**0.0295**		
Bright erythema				
Time	1.19 (1.15–1.23)	** < 0.0001**	1.21 (1.17–1.26)	** < 0.0001**
Gender	0.41 (0.23–0.71)	**0.0012**	0.12 (005–0.27)	** < 0.0001**
Epilation				
Time	1.30 (1.24–1.38)	** < 0.0001**		
Dry desquamation				
Time	1.04 (1.02–1.07)	**0.0001**	1.04 (1.02–1.07)	**0.0001**
Gender	0.28 (0.07–0.74)	**0.0084**	0.26 (007–0.71)	**0.0064**
Moist desquamation				
Time	1.31 (1.24–1.41)	** < 0.0001**	1.32 (1.24–1.42)	** < 0.0001**
Radiochemotherapy	1.82 (1.00–3.50)	**0.0497**	0.51 (0.19–1.33)	0.1701
Moderate edema				
Time	1.13 (1.10–1.17)	** < 0.0001**		
Pitting edema				
Time	1.19 (1.13–1.27)	** < 0.0001**		
Ulceration				
Time	1.09 (1.03–1.18)	**0.0012**		

Significant values are in [bold].

**Figure 2:**
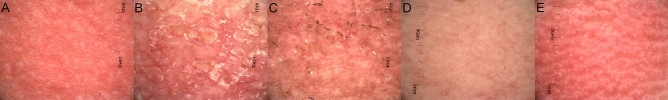
Dermoscopic images of ARD described in line with the consensus of experts in non-neoplastic dermatoses on behalf of the International Dermoscopy Society by Errichetti et al.^17^. (**A**) Dermoscopic image of ARD reveals linear branched, linear curved vessels with reticular distribution and perifollicular white color; (**B**) dermoscopic image of acute radiodermatitis reveals white and yellow, patchy scale; (**C**) dermoscopic image reveals linear branched and linear curved vessels with clustered distribution, brown, patchy scale; (**D**) dermoscopic image reveals linear branched and linear curved vessels with unspecific distribution, and perifollicular pigmentation; (**E**) linear branched and linear curved vessels with reticular distribution and follicular plugs arranged in rosettes.

Based on the results in Table 4, we observed the relationship between the presence of vessels in reticular distribution and time, age, gender, induction chemotherapy, and concurrent CHT (Table 4). The statistical interpretation of the OR (univariate regression) may be as follows: 1 day of observation generates an increased risk of vessels in reticular distribution by 8%, and 5 days of observation (1.08^5^ = 1.47), so by almost one and a half. A 10-year difference in the age of patients generates a (1–0.97^10^) × 100% = 26% reduction in the occurrence of vessels in reticular distribution. The risk of vessels in reticular distribution is 64% lower in men than in women. Induction chemotherapy reduces the risk of vessels in reticular distribution almost three times (OR = 2.94). Concurrent CHT reduces the risk of vessels in reticular distribution by 1.83 (OR = 1.83). The results regarding the effect of collected risk factors on skin reaction in a multivariate model showed that the effect of gender and induction chemotherapy increased. Moreover, in the multivariate model, the lack of concurrent CHT reduces the risk of vessels in reticular distribution by 53% (see the right panel of Table 4). Other results in the table should be interpreted analogously. Considering individual factors affecting clinical response, each day of observation during RT treatment statistically generates a higher chance of occurrence of vessels in reticular distribution (Figs. 1D,F,H, 2A,E), white scale and yellow scale (Fig. 2B), and brown scale (Fig. 2C) with patchy distribution (Figs. 1B,H, 2C), perifollicular pigmentation (Fig. 2D), follicular plugs arranged in rosettes (Fig. 2E), while the chance of unspecific distribution of vessels decreases (Fig. 2D). In the context of a macroscopic response, each day of observation during RT treatment statistically generates a higher chance of occurrence of bright erythema (Fig. 1C,E), epilation (Fig. 1C,E), dry and moist desquamation (Fig. 1C,E), moderate (Fig. 1C) and pitting edema (Fig. 1E), and ulceration (Fig. 1G) while the chance of follicular and faint erythema decreases (Fig. 1A). The results regarding the effect of collected risk factors on skin reaction in a multivariate model were comparable. In a univariate analysis, age was a significant factor for vessels in reticular distribution, vessels in unspecific distribution, follicular plugs arranged in rosettes, and perifollicular pigmentation as well as in the group of macroscopic features for follicular erythema. However, multivariate analysis did not show this relationship for the unspecific distribution of vessels, follicular plugs arranged in rosettes, or perifollicular pigmentation (the association is on the border of statistical significance, i.e., p < 0.1). Gender is important for the occurrence of vessels in reticular distribution, vessels in unspecific distribution, white, yellow, patchy scale, follicular plugs arranged in rosettes, and for macroscopic features for faint and bright erythema and dry desquamation. Multivariate analysis showed that the gender effect was stronger in each case. The risk of vessels in reticular distribution, white scale, yellow scale, patchy scale, and follicular plugs arranged in rosettes is 85%, 89%, 80%, 95%, and 69% lower in men than in women, respectively. The risk of faint erythema is 118% higher for men than women, while the chance of bright erythema and dry desquamation is 88% and 74% lower in men than in women, respectively. Induction chemotherapy increases the risk of yellow scale and reduces the risk of vessels in reticular distribution. In the multivariate model, these dependencies increase, and we observe that induction chemotherapy increases the risk of yellow scale occurrence by two-thirds (OR = 0.34) and reduces the risk of vessels in reticular distribution almost six times (OR = 5.90). Concurrent chemotherapy is important for the occurrence of vessels in reticular distribution, follicular plugs arranged in rosettes, perifollicular pigmentation and macroscopic features such as follicular erythema, tender erythema, and moist desquamation. In turn, multivariate analysis did not show this relationship for follicular plugs arranged in rosettes, follicular erythema and moist desquamation (the association is on the border of the statistical significance, i.e., p < 0.1). Non-concurrent chemotherapy reduces the risk of vessels in reticular distribution, perifollicular pigmentation and tender erythema by 53%, 51% and 94%, respectively.

## Discussion

Graham et al. emphasized the importance of archiving photographs, which are a useful source of documents for auditing and monitoring radiotherapy-induced skin toxicity^18^. In turn, the study by Ni et al. used deep learning-based method for the automatic assessment of radiation-induced dermatitis in patients with nasopharyngeal carcinoma^19^. In our study, 2244 dermoscopic photographs and 374 clinical photographs were archived, creating a database that in the future could be used as a database to automate clinical assessment. In the current literature, only one study used dermoscopy, but only for the presence of erythema in ARD^20^. So far, only clinical features have been described, and there are no data on the analysis of dermoscopic features in ARD. One of the previous studies reported dermoscopic changes in the surrounding tissue of basal cell carcinoma in patients who underwent brachytherapy^21^. Radiation-induced dermatitis occurs in about 90–95% of patients exposed to ionizing radiation^22–24^. Published reports on the share of individual grades per RTOG are ambiguous. This is probably due to many variables affecting the development of this type of skin toxicity. Elliot et al. showed in their observation that 1% of patients did not develop any grade of ARD, 20% developed grade I, 57% grade II, and 23% grade III or IV^25^. Kang et al. observed radiation-induced dermatitis of the maximum grade I-IV in 46.6%, 18.0%, 5.5%, and 0.9% of the patients, respectively^26^. In turn, in the report from Franco et al., the toxicity profile at the end of RT was Grade 0 in 3.5% of patients, Grade I in 32%, Grade II in 61%, Grade III in 3.5%^27^. Mild erythema may appear as early as a few hours after exposure to ionizing radiation^28^, but usually develops about 7–10 days after starting therapy^29^. Dry desquamation (RTOG/EORTC grade I) usually occurs after 3–4 weeks from the start of treatment. More intense erythema, hair loss, and hyperpigmentation are usually observed between 2 and 4 weeks of therapy^30^. Moist desquamation (RTOG/EORTC grade II) usually occurs after 4 weeks when the total RT dose to the skin is 40 Gy or higher^31,32^. In the study of Franco et al., grade II appeared between treatment weeks 4–5; for those having grade III acute skin toxicity, this event mainly began during weeks 5 and 6^27^. Data variability is also likely to be influenced by treatment and clinical risk factors. ARD can lead to pain, discomfort, reduced quality of life, and premature discontinuation of treatment. Therefore, it is important to make a rapid diagnosis when the first symptoms appear and to implement appropriate prevention and treatment. Dermoscopy can be a complementary tool to support macroscopic ARD evaluation. Our study is the first in the published papers to attempt to identify the correlations between the clinical and dermoscopic features of ARD with its dermoscopic follow-up. The importance of the total dose during RT is well known^33,34^. Moreover, in our study, we selected patients scheduled for RT at comparable total doses to minimize the risk of a dose effect. A statistical dependence of the influence of days of observation during RT was observed for the features correlating in the test of compatibility of clinical and dermoscopic features. Predicting the risk of radiation-induced dermatitis is essential for proper prevention and treatment. Kawamura et al. in their study created a scoring system taking into account V60Gy, concurrent chemotherapy status, age, and body mass index^35^. Age ≥ 67 years was significant in their study for the development of ARD.

Meyer et al. showed that gender is important in the context of the development of radiation-induced dermatitis^33^. Kawamura et al. showed that concurrent chemotherapy with platinum and cetuximab (cetuximab > platinum) had significant importance in the development of radiation-induced dermatitis. Gold standards of management have not yet been established, and treatment, as well as prevention, are common and empirical, based on personal experience supported by weak scientific evidence^9,36^. Based on the study by Robijn et al., there could be a strong recommendation to use photobiomodulation therapy (PBMT) in the prevention and management of ARD in cancer patients^37^. The identified dermoscopic features may facilitate the selection of topical preparations in further studies, which will consider dermoscopic image in the skin and facilitate non-invasive adjustment of prophylaxis and treatment of ARD. Because very frequent observations of patients showed the appearance of the first features on average on 4.69 days from the first dose of ARD, appropriate prevention should be implemented rapidly, especially in males, who in a recent study were found to develop a higher degree of ARD.

### Conclusions (PURE)

Knowledge of dermoscopic features and predictors could help in rapid early assessment and new therapeutic strategies, that can help reduce toxicities among patients treated with RT for HNC.

## Data Availability

The datasets used and/or analysed during the current study available from the corresponding author on reasonable request.

## Abbreviations

HNC: Head and neck cancer
RT: Radiotherapy
ARD: Acute radiation-induced dermatitis
AJCC: American Joint Committee on Cancer
CTV1: Clinical target volume 1
PTV1: Planned target volume 1
RTOG/EORTC: Radiation Therapy Oncology Group and the European Organization for Research and Treatment of Cancer
CTCAE: Common Terminology Criteria for Adverse Events
LENT SOMA: Late Effects Normal Tissue Task Force-Subjective scale, and the Objective, Management, Analytic scale
indCHT: Induction chemotherapy
CHRT: Radiochemotherapy
IDS: International Dermoscopy Society
OR: Odds ratio
CI: Confidence interval
PBMT: Photobiomodulation therapy
